# Novel fusion proteins for the antigen-specific staining and elimination of B cell receptor-positive cell populations demonstrated by a tetanus toxoid fragment C (TTC) model antigen

**DOI:** 10.1186/s12896-016-0249-x

**Published:** 2016-02-17

**Authors:** Diana Klose, Ute Saunders, Stefan Barth, Rainer Fischer, Annett Marita Jacobi, Thomas Nachreiner

**Affiliations:** Department of Experimental Medicine and Immunotherapy, Institute for Applied Medical Engineering, University Hospital RWTH Aachen, Pauwelsstr. 20, 52074 Aachen, Germany; Department of Internal Medicine D, Rheumatology and Clinical Immunology Unit, University Hospital Münster, Domagkstr. 3, 48149 Münster, Germany; Department of Pharmaceutical Product Development, Fraunhofer-Institute for Molecular Biology and Applied Ecology IME, Forckenbeckstr. 6, 52074 Aachen, Germany; Institute for Molecular Biotechnology, RWTH Aachen University, Worringer Weg 1, 52074 Aachen, Germany; South African Research Chair in Cancer Biotechnology, Institute of Infectious Disease and Molecular Medicine (IDM), Department of Integrative Biomedical Sciences, Faculty of Health Sciences, University of Cape Town, Anzio Road, 7925 Observatory, South Africa; Division of Rheumatology and Clinical Immunology, Brandenburg Medical School, Fehrbelliner Str. 38, 16816 Neuruppin, Germany

**Keywords:** B cell receptor, Targeted therapy, Recombinant fusion protein, Periplasmic protein expression, Tetanus toxoid fragment C, Memory B cells

## Abstract

**Background:**

In an earlier study we developed a unique strategy allowing us to specifically eliminate antigen-specific murine B cells via their distinct B cell receptors using a new class of fusion proteins. In the present work we elaborated our idea to demonstrate the feasibility of specifically addressing and eliminating human memory B cells.

**Results:**

The present study reveals efficient adaptation of the general approach to selectively target and eradicate human memory B cells. In order to demonstrate the feasibility we engineered a fusion protein following the principle of recombinant immunotoxins by combining a model antigen (tetanus toxoid fragment C, TTC) for B cell receptor targeting and a truncated version of *Pseudomonas aeruginosa* exotoxin A (ETA’) to induce apoptosis after cellular uptake. The TTC-ETA’ fusion protein not only selectively bound to a TTC-reactive murine B cell hybridoma cell line in vitro but also to freshly isolated human memory B cells from immunized donors ex vivo. Specific toxicity was confirmed on an antigen-specific population of human CD27^+^ memory B cells.

**Conclusions:**

This protein engineering strategy can be used as a generalized platform approach for the construction of therapeutic fusion proteins with disease-relevant antigens as B cell receptor-binding domains, offering a promising approach for the specific depletion of autoreactive B-lymphocytes in B cell-driven autoimmune diseases.

**Electronic supplementary material:**

The online version of this article (doi:10.1186/s12896-016-0249-x) contains supplementary material, which is available to authorized users.

## Background

B-lymphocytes play a major role in humoral immunity and alterations in B cell development can cause certain types of autoimmune diseases [1]. The secretion of self-reactive antibodies by B-lymphocytes significantly contributes to disease pathogenesis [2, 3]. In addition to the secretion of autoantibodies, self-reactive B cells also present autoantigens on their surface to stimulate pathogenic T cells. In autoimmune diseases, the abnormal recognition of autoantigens by self-reactive B cells and T cells leads to severe tissue damage. Moreover, autoreactive B-lymphocytes are characterized by a deregulated cytokine milieu reflecting the production of proinflammatory cytokines such as interleukin-6 (IL-6), interferon-gamma (IFN-γ), IL-4 and TGF-beta, all of which can trigger dendritic cell migration, macrophage activation or affect the regulatory role of T cells [4, 5]. Common strategies for the treatment of autoimmune diseases include the use of immunosuppressive agents such as steroids, cytotoxic drugs or immunomodulatory agents to restore homeostasis in the immune system and depress the autoimmune response [2, 6]. These therapeutic agents offer symptomatic relief by inhibiting general inflammatory responses, but they do not exclusively target the misdirected immune cells that are causing the disease. Many standard therapeutic approaches affect all immune cells, regardless of specificity, function or activation status. Current therapeutic strategies aim to eliminate various pathogenic cell populations without targeting self-reactive memory B cells directly [1, 7, 8]. With the help of a model antigen well characterized in the human system we aim to demonstrate the antigen-specific targeting of human B-lymphocytes via their B cell receptor (BCR), which can offer a novel and promising alternative approach for the treatment of autoimmune diseases [9, 10]. We used protein engineering to generate fusion proteins consisting of a cell-specific binding domain (an antigen or a fragment thereof) fused to an effector domain (cytolytic or cytotoxic protein). The most potent recombinant immunotoxins today are based on truncated versions of *Pseudomonas aeruginosa* exotoxin A (ETA’) [9–12]. The tetanus toxoid fragment C (TTC) is often used as a model antigen because many people worldwide are vaccinated with tetanus toxoid, and the well-established TTC fragment is characterized by a frequency of 0.01 % TTC-reactive memory B cells within the B cell pool without a recent booster vaccination [13]. The first requirement for a functional toxic fusion protein is the specific binding to the BCR of self-reactive B cells, followed by receptor-mediated internalization, the release of the catalytic moiety from the endosomes for intracellular transport from the Golgi into the endoplasmic reticulum, and finally its cytosolic release. This allows ETA’ to exert its cytotoxic activity via ADP-ribosylation of eukaryotic elongation factor 2 (eEF2), leading to efficient inhibition of protein synthesis and ultimately to apoptosis [14, 15]. The new fusion protein undergoes rapid receptor-mediated endocytosis via the BCR [16]. We generated a TTC-ETA’ fusion protein for the specific depletion of TTC-reactive B-lymphocytes isolated from human blood. For straightforward staining purposes of TTC-specific cell populations we developed a TTC-SNAP-tag fusion protein allowing the covalent coupling of the fusion protein to benzylguanine-conjugated fluorescent dyes to examine binding kinetics at B cell surfaces [17]. Even if expressed in two different expression systems, both proteins bound specifically to TTC-reactive cells with similar binding characteristics. Further, the TTC-ETA’ fusion protein demonstrated specific cytotoxicity towards human TTC-reactive memory B cells ex vivo. The results of previous investigations performed by Volkman et al. suggested that human TT-antibody responses can be inhibited specifically in vitro using a TT-ricin conjugate. Using a modified approach and a more elaborated read out this work aims to confirm and quantify the selective depletion of human TTC-specific memory B cells by an antigen-ETA’ fusion protein. Based on the results of this study, we believe that this concept has a platform character and can be applied to generate powerful fusion proteins for immunotherapeutic approaches.

## Methods

### Cloning of expression vectors

**Fig. 1:**
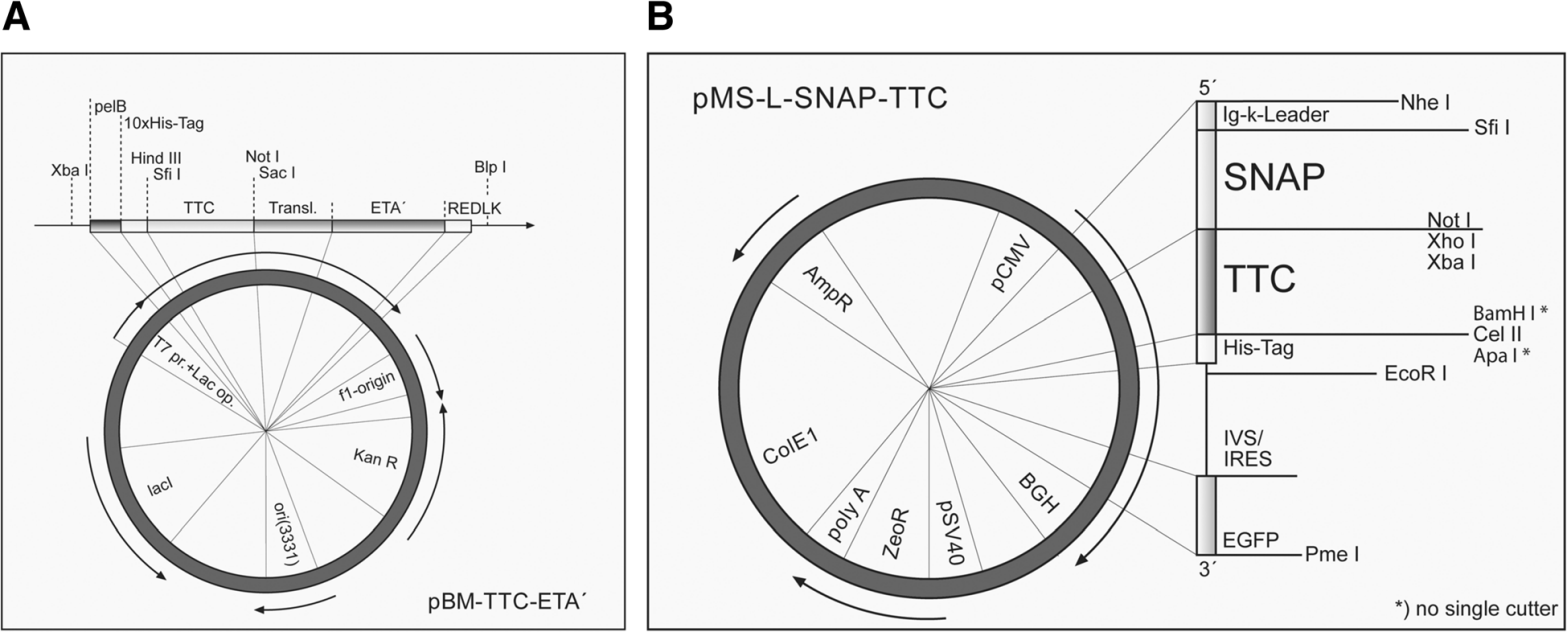
Illustration of the prokaryotic and eukaryotic expression vectors. The synthetic tetanus toxoid fragment C (TTC) DNA sequence was cloned into the prokaryotic expression vector pBM and the eukaryotic expression vector pMS using the restriction sites *Sfi*I and *Not*I. **a** Prokaryotic expression vector pBM-TTC-ETA’. pelB = signal sequence for protein secretion into the periplasm; 10xHis-tag = polyhistidin-sequence for detection and purification of recombinant proteins; A = antigen fragment; ETA’ = deletion mutant of *Pseudomonas aeruginosa* exotoxin A; f1 ori = origin of replication for production of single-stranded DNA by M13-helper phage; kanR = kanamycin resistance gene for he selection of transformed cells; ori(3331) = origin of replication; lacI = Lac repressor; T7 prom + Lac op. = IPTG-inducible promotor + Lac-Operator. **b** Eukaryotic expression vector pMS-L-SNAP-TTC. pCMV = constitutive active promotor of the cytomegalovirus; Ig-κ-Leader = murine signal sequence for protein secretion into the cell culture supernatant; Myc/His-tag = c-myc-epitope for detection/polyhistidin-tag for detection and purification; eGFP = enhanced green fluorescent protein; BGH = Bovine growth hormone (BGH) polyadenylation signal, ZeoR = Zeocin® resistance gene for the selection of transfected cells, pSV40 = early SV40-promotor, SV40 replication origin (ORI); polyA = polyadenylation signal, ColE1 origin = bacterial origin of replication; AmpR = ampicillin resistance gene for the selection of transformed *Escherichia coli*

The tetanus toxoid fragment C (TTC) DNA sequence (GenBank accession no. FJ917402.1) was synthesized by GeneArt® Gene Synthesis (Life Technologies, Darmstadt, Germany) and included the restriction sites *Sfi*I and *Not*I. After digestion with these enzymes, the TTC DNA sequence was ligated into the *Sfi*I*/Not*I sites of pBM1.1 with and without the ETA’ sequence [18] and pMS [19] (Fig. 1). Successful cloning was confirmed by colony polymerase chain reaction and DNA sequencing.

### Expression of TTC-ETA’ and TTC in Escherichia coli and protein purification

*Escherichia coli* BL21 (DE3) cells (Novagen, Darmstadt, Germany) were transformed with the TTC and TTC-ETA’ encoding expression vectors and the corresponding proteins were expressed into the periplasm under osmotic stress in the presence of compatible solutes [20]. The protein was purified from the periplasmic fraction by immobilized metal-ion affinity chromatography (IMAC) using a Nickel-Sepharose (Ni-NTA) Superflow Cartridge (Qiagen, Hilden, Germany) on the ÄKTApurifier system (GE Healthcare Life Sciences, Freiburg, Germany) followed by a size-exclusion chromatography using a Superdex 200 (GE Healthcare). The TTC proteins were eluted into phosphate buffered saline (PBS, pH 7.4) and concentrated using Vivaspin 6 columns (Sartorius, Goettingen, Germany). The proteins were passed through a 0.22-μm sterile filter (Nalgene, Roskilde, Denmark) and analyzed by sodium dodecylsulfate polyacrylamide gel electrophoresis (SDS-PAGE) as previously described [21]. After gel staining with Coomassie Brilliant Blue, the protein concentration was estimated using AIDA Image Analyzer (Raytest GmbH, Straubenhardt, Germany) against 0.25-3 μg standards of bovine serum albumin (BSA). Unstained gels were blotted onto nitrocellulose membranes and probed with a primary anti-penta-his antibody diluted 1:5000 (Qiagen, Hilden, Germany; Catalogue number: 34660) and an alkaline phosphate (AP)-coupled goat-anti-mouse secondary antibody also diluted 1:5000 (Dianova, Hamburg, Germany; Catalogue number: 115-055-020).

### Expression of SNAP-TTC in HEK293T cells

Human embryonic kidney cells (HEK293T, ATCC, Wesel, Germany, CRL-11268) were cultured under standard conditions (RPMI 1640 medium, 10 % v/v fetal calf serum (FCS), 100 U/ml penicillin, 100 mg/ml streptomycin, 37 °C, 5 % CO_2_) and transfected with the pMS expression vectors using Roti®-Fect (Carl Roth, Karlsruhe, Germany) according to the manufacturer’s instructions. Briefly, 2 μg of DNA was mixed with 2 μl of Roti®-Fect and applied to the cells for 3 h. The cultures were then supplemented with 100 ng/ml Zeocin® (Invitrogen, Carlsbad, USA) for selection. The supernatant was collected from the transfected HEK293T cells and the protein was purified by IMAC as described above. After overnight dialysis against 1 x PBS at 4 °C, the SNAP-TTC protein was passed through a 0.22-μm sterile filter (Nalgene, Roskilde, Denmark). The protein concentration was determined using the AIDA Image Analyzer as described above.

### Coupling SNAP-TTC to the fluorescent dye

Purified SNAP-TTC was conjugated to the BG-modified fluorescent dyes SNAP-surface® Alexa Fluor® 647 (New England Biolabs, Frankfurt am Main, Germany; Catalogue number: S9136S) and SNAP-surface® Alexa Fluor® 488 (New England Biolabs; Catalogue number: S9129S) as previously described [17]. Briefly, 1 μg SNAP-TTC protein was mixed with 2 nmol BG-647 or BG-488 solution prepared from a 50 nmol stock and incubated for 1 h at room temperature. Samples were taken to determine the coupling efficiency by SDS-PAGE and the fluorescence signal was detected using the CRi Maestro imaging system with appropriate filter sets (Perkin Elmer, Waltham, MA, USA).

### Cultivation of hybridoma cells and cell binding analysis by flow cytometry

TTC-reactive 5E4 hybridoma cells (kindly provided by Prof. Dr. M. Shapiro, Rockville, USA) and myelin oligodendrocyte glycoprotein (MOG)-reactive 8.18-C5 hybridoma cells (kindly provided by Prof. C. Linington, Glasgow, UK) were cultured under standard conditions (RPMI 1640 medium, 10 % FCS, 100 U/ml penicillin, 100 mg/ml streptomycin, 37 °C, 5 % CO2). The binding of TTC fusion proteins to 5 × 10^5^ TTC-reactive hybridoma cells was evaluated by flow cytometry using a BD FACSVerse flow cytometer (Becton Dickinson, Heidelberg, Germany) and BD FACSuite analysis software. Briefly, the cells were incubated with 100 nM purified TTC fusion proteins for 20 min and then with 1 μg/ml anti-His5 AlexaFluor 488 antibody (Cat. no. 35310, Qiagen, Hilden, Germany) for 20 min, in each case on ice with intermediate washes in 1.8 ml 1 x PBS. Dose-dependent binding of TTC-ETA’ was analyzed on TTC-reactive 5E4 hybridoma cells. MOG-reactive 8.18-C5 hybridoma cells were used as a negative control. Briefly, 2 x 10^5^ target cells were incubated with various concentrations (1–400 nM) of TTC-ETA' followed by incubation with 1 μg/mL Penta-His Alexa Fluor 488 Conjugate antibody (Cat. no. 35310, Qiagen, Hilden, Germany). Cells were washed twice with 1x PBS in each step and finally eluted in FACS buffer. The binding activity of TTC-ETA' was analyzed on a BD FACSVerse flow cytometer (Becton Dickinson) using the BD FACSuite analysis software (Becton Dickinson). Mean values of three independent measurements were normalized and fitted using the GraphPad Prism v5 software (GraphPad Software, Inc., La Jolla, CA, USA).

### Hybridoma cell viability assay

Hybridoma 5E4 and 8.18-C5 cells (1 x 10^4^ cells/well) were incubated with serially diluted TTC-ETA’ and TTC proteins in cell culture media (RPMI 1640, 10 % v/v FCS) at 37 °C and 5 % CO2 for 72 h. Cell viability was determined by adding XTT/phenazine methosulfate in 50 μl RPMI medium and incubating as above for 4 h. Absorbance was measured at 450 nm and 630 nm (reference wavelength) on a BioTek ELISA reader (Bad Friedrichshall, Germany). Results are presented as the means of three independent experiments with the corresponding standard deviations. The half maximal inhibitory protein concentration (EC50) was calculated relative to untreated control cells. Raw data were fitted using the GraphPad Prism v5 software (GraphPad Software, Inc., La Jolla, CA, USA).

### Isolation of B-lymphocytes

For enzyme-linked immunospot (ELISPOT) assays CD27^+^ memory B cells were isolated from leukocyte filters obtained from tetanus-vaccinated blood donors. The local ethics committee approved this procedure. The donors gave written informed consent and were asked for their status of vaccination. First, total B cells were isolated by negative selection using the RosetteSep™ Human B Cell Enrichment Cocktail (STEMCELL Technologies SARL, Grenoble, France) and subsequent density gradient centrifugation with Ficoll-Paque PLUS (GE Healthcare, Munich, Germany). After two washing steps with PBS containing 0.5 % BSA (Sigma-Aldrich, Taufkirchen, Germany) the B cells were immediately labeled with CD27 MicroBeads (Miltenyi Biotec, Bergisch Gladbach, Germany; Catalogue number: 130-051-601) and subsequently isolated using LS columns and a MidiMACS Separator according to the manufacturer’s instructions (Miltenyi Biotec). Thereafter, the isolated CD27^+^ memory B cells were incubated at a concentration of 10^5^ cells per ml in RPMI 1640 medium supplemented with 10 % v/v FCS, 100 U/ml penicillin and 100 mg/ml streptomycin (all from Life Technologies GmbH, Darmstadt, Germany) on a 96-well round-bottomed plate (Greiner Bio-One, Frickenhausen, Germany) with 2.5 μg/ml CpG oligodeoxynucleotide 2006 (5’-TCG TCGTTTTGTCGTTTTGTCGTT-3’, TIB MolBiol, Berlin, Germany) and 50 ng/ml interleukin (IL)-21 (Life Technologies GmbH, Darmstadt, Germany). After polyclonal activation with the TLR-9 agonist CpG and IL-21, 10 nM TTC or TTC-ETA was added to the culture medium on day 2. On day 4, activated cells were harvested and ELISPOT assays were carried out to determine the frequencies of IgG, anti-tetanus toxoid (TT) IgG as well as anti-TTC specific IgG antibody secreting cells (ASCs). For flow cytometric analyses CD19 MicroBeads (Miltenyi Biotec; Catalogue number: 130-097-055) instead of CD27 MicroBeads were used for positive selection and peripheral blood mononuclear cells (PBMCs) were isolated from a donor 6 and 14 days after booster vaccination with tetanus toxoid to determine the binding of the TTC fusion protein to ex vivo plasma cells and memory B cells, respectively. Briefly, PBMCs were isolated by density gradient centrifugation with Ficoll-Paque PLUS (GE Healthcare) and stained for TTC-specific CD27^++^CD38^++^ plasma cells or CD27^+^ memory B cells.

### Binding analysis of the TTC fusion protein to B-lymphocytes by flow cytometry

The staining was performed with the following monoclonal antibodies against the human B cell specific surface markers CD19 (Becton Dickinson; Catalogue number: 560911), CD27 (Becton Dickinson; Catalogue number: 337169), CD38 (Becton Dickinson, Catalogue number: 345806) and biotinylated IgD (Becton Dickinson; Catalogue number: 555777) in combination with streptavidin Alexa-Fluor 680 (Life Technologies; Catalogue number: S21378) in combination with the recombinant TTC protein followed by an Alexa Fluor 488-conjugated anti-penta-his antibody (Qiagen; Catalogue number: 35310). For comparison, a FITC-labeled TTC peptide (List Biological Laboratories, Inc., CA, USA) was used. For the intracellular TTC staining saponin was added. In the case of surface staining dead cells were excluded by labeling with 220 nM 4’,6-diamidino-2-phenylindole (DAPI; Life Technologies). Flow cytometry was carried out using a FACSCanto™ with FACS DIVA Software (Becton Dickinson) and the results were analyzed using the FlowJo software (Treestar, Ashland, OR, USA).

### Quantitation of TT- and TTC-specific ASCs by ELISPOT after polyclonal activation of CD27^+^ memory B cells

After polyclonal activation with the TLR-9 agonist CpG and IL-21 for 4 d, we carried out ELISPOT assays to determine the frequency of IgG^+^ anti-tetanus toxoid (TT) and anti-TTC specific ASCs. Based on the results of the hybridoma cell viability assay, on day 2 of cultivation, the memory B cells were treated with 10 nM TTC or TTC-ETA’. To determine the frequencies of total ASCs, high-binding 96-well ELISA plates (Greiner Bio-One) were pre-coated overnight at 4 °C with goat anti-human kappa and lambda antibodies (SouthernBiotech, AL, USA; Catalogue numbers: SBA1050-04, SBA1060-04). To determine the frequencies of tetanus-specific ASCs, high-binding 96-well ELISA plates were pre-coated with 20 Lf/ml tetanus toxoid (Statens Serum Institute, Copenhagen, Denmark) or with 20 μg/ml TTC protein. After three washes with 150 μl/well PBS plus 0.5 % BSA, the plates were blocked with 200 μl/well PBS containing 3 % BSA and 5 % FCS for 1 h at 37 °C in a 5 % CO2 atmosphere. The obtained cells were titrated and plated in duplicates in the following 10 dilutions: 5–1,000 cells/well for total IgG and 2,000-100,000 cells/well for TT and TTC IgG ELISPOTs. After incubating for 8 h at 37 °C, the plates were washed six times with 150 μl/well PBS. Spots were visualized using AP-conjugated goat anti-human IgG (SouthernBiotech; Catalogue number: SBA1030-04) for 1 h at 37 °C in a 5 % CO2 atmosphere, followed by adding the 2-amino-2-methyl-1-propanol (AMP) buffer supplemented with 5-bromo-4-chloro-3-indolyl-phosphate (BCIP)/nitroblue tetrazolium (NBT). After counting the spots, the frequencies of TT and TTC IgG-secreting ASCs were calculated for each individual.

### Statistical analysis

Statistical analyses were based on at least three independent series of experiments in triplicates. The two-tailed Student’s *t-*test was used for statistical analysis. A P value of less than 0.05 was considered statistically significant. Calculations were performed using GraphPad Prism v5 software.

## Results

### Expression of TTC fusion proteins

**Fig. 2:**
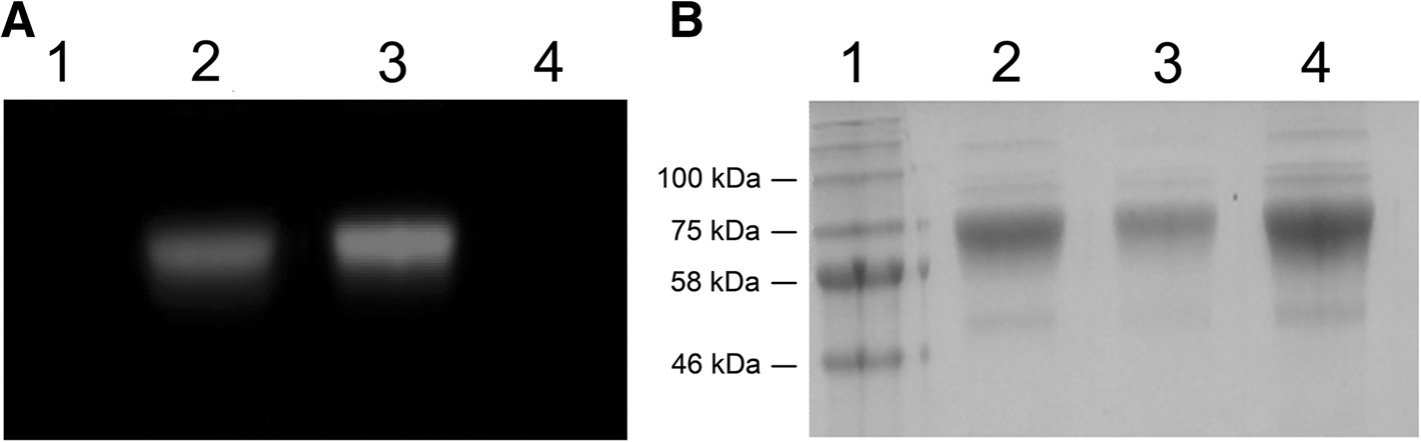
Fluorescent in-gel detection of SNAP-TTC labeled with different dyes. **a** SDS-PAGE of SNAP-TTC fusion protein labeled with SNAP-Surface® Alexa Fluor® 488 (2) or BG-647 (3), respectively. Fluorescence signals were visualized using the Maestro CRi in vivo imaging system with the appropriate filter set. **b** Coomassie-stained SDS gel from (**a**). The stained protein bands correspond to the measured fluorescence signals from (**a**). (1) prestained protein marker, broad range (NEB), (2) SNAP-TTC-SNAP-Surface® Alexa Fluor® 488, (3) SNAP-TTC-BG647, (4) uncoupled SNAP-TTC protein

The prokaryotic and eukaryotic expression vectors containing the TTC cell-binding domain were sequenced to verify the correct format of the fusion domains. The pBM expression vectors (Fig. 1a) were then introduced into *Escherichia coli* strain BL21 (DE3) and the pMS expression vector (Fig. 1b) was used for the transient transfection of HEK293T cells. TTC and TTC-ETA’ proteins were periplasmically expressed in *E. coli* BL21 (DE3) cells under osmotic stress conditions in the presence of compatible solutes. After purification by IMAC using a Ni-Sepharose Superflow cartridge followed by size exclusion chromatography, we achieved yields of up to 1.1 mg TTC (MW: 55 kDa) and 1.8 mg TTC-ETA’ (MW: 95 kDa) per liter of bacterial culture. The purified fusion proteins were analyzed by SDS-PAGE and western blot using a mouse anti-His6 monoclonal antibody (Additional file 1: Figure S1). The SNAP-TTC fusion protein expressed in transfected HEK293T cells was secreted into the cell culture supernatant and purified by IMAC using a Ni-Sepharose Superflow cartridge as above, resulting in a yield of 6 mg per liter of cell culture supernatant. The protein was analyzed by SDS-PAGE and the corresponding western blot confirmed the expression of SNAP-TTC (data not shown). The SNAP-TTC protein was successfully coupled to the fluorescent dyes BG-488 or BG-647 and detected using the CRi Maestro imaging system (Fig. 2a). The SDS polyacrylamide gel was then stained with Coomassie Brilliant Blue and we confirmed that the protein bands matched the corresponding fluorescence signals (Fig. 2b).

### Binding of TTC and TTC-ETA’ to hybridoma cells

**Fig. 3:**
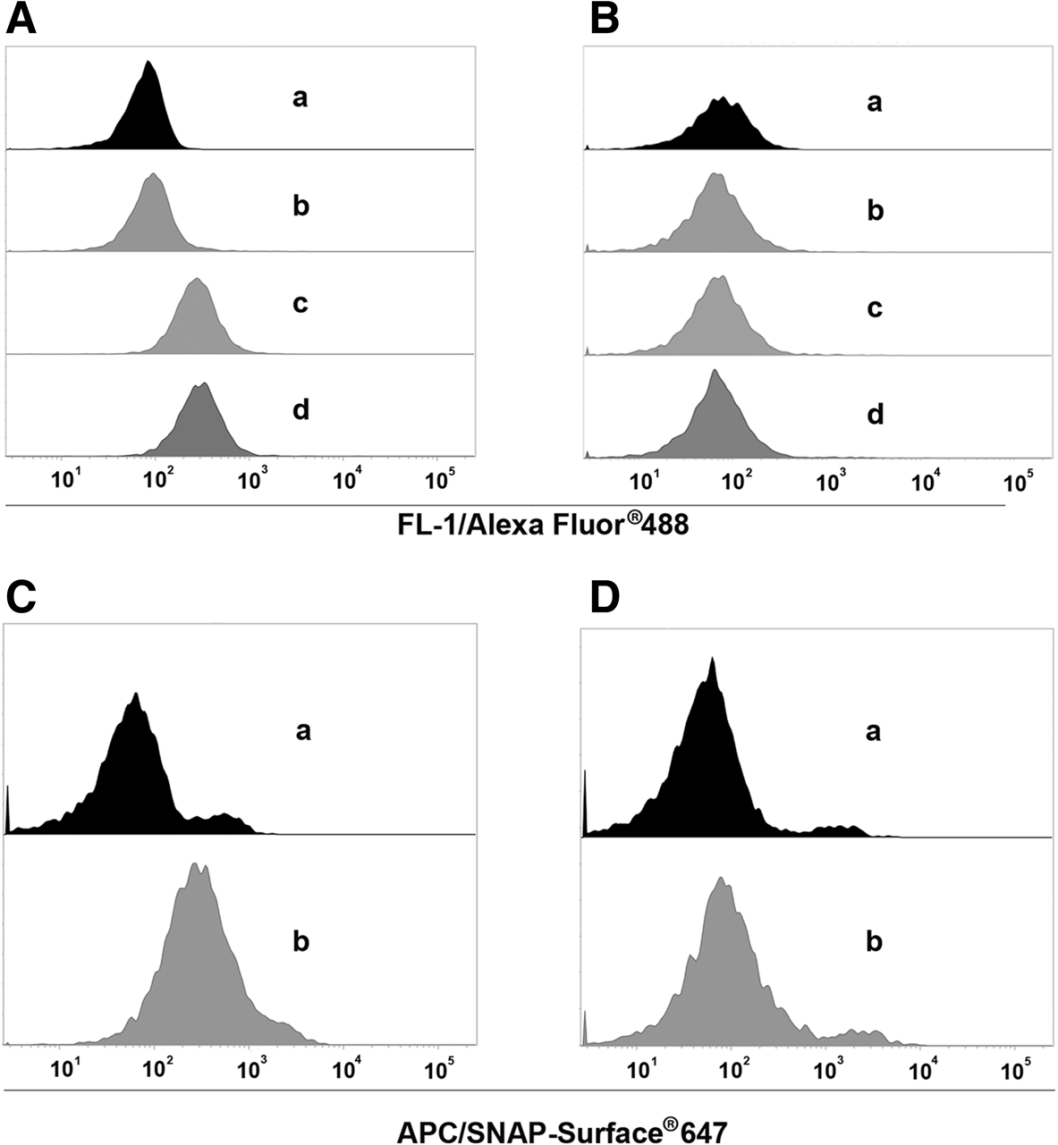
Binding analysis of recombinant TTC-based proteins to TTC-reactive hybridoma cells. Equimolar amounts (100 nM) of TTC (*c*) and TTC-ETA’ (*d*) were used for binding analysis to the TTC-reactive hybridoma cell line 5E4 (**a**) compared to the control hybridoma cell line 8.18-C5 (**b**). Detection of bound proteins was carried out using an Alexa Fluor® 488-coupled anti-His5 antibody. Staining with Alexa Fluor 488-coupled anti-His5 antibody (*b*) and unstained cells (*a*) served as controls. Binding analysis of 100 nM SNAP-TTC coupled to the SNAP-Surface® 647 fluorescence dye (*b*) to 5E4 hybridoma cells (**c**) and to the control hybridoma cell line 8.18-C5 (**d**). Unstained cells served as control (*a*)

**Fig. 4:**
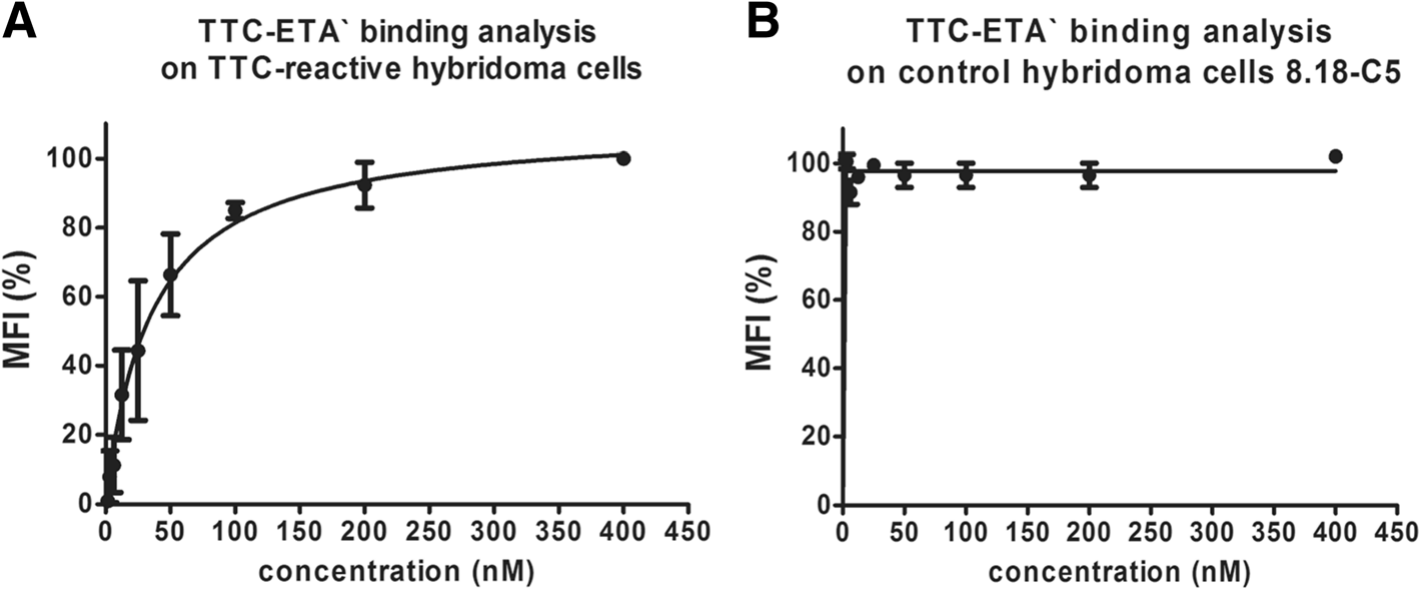
Dose-dependent binding analysis of the recombinant fusion protein TTC-ETA’ on hybridoma cells. Various concentrations (1–400 nM) of TTC-ETA’ were used to determine a dose-dependent binding activity on TTC-reactive hybridoma cell line 5E4 (**a**) and to exclude specific binding to the control hybridoma cell line 8.18-C5 (**b**). The detection of bound protein was carried out by flow cytometry using a Penta-His Alexa Fluor 488 Conjugate antibody. Measurements were performed in triplicates (n = 3); error bars indicate SD. The recombinant TTC-ETA’ exhibits a dosedependent binding on the target hybridoma cell line 5E4, whereas no binding could be determined on the control hybridoma cell line 8.18-C5

TTC-ETA’ selectively targets TTC-reactive hybridoma cells and TTC-reactive memory B cells by binding to the TTC-reactive BCR. This binding activity is necessary for the biological function of the protein and was confirmed by investigating the ability of the fusion protein to bind TTC-reactive 5E4 hybridoma cells, which express the membrane-bound form of the BCR. The quantity of protein bound to the cell surface was determined by flow cytometry using an Alexa Fluor 488-conjugated anti-penta-his antibody. This confirmed that both TTC and TTC-ETA’ bound to the TTC-reactive hybridoma cells, whereas no binding was observed on the control cell line (MOG-specific hybridoma 8.18-C5 cells) as shown in Fig. 3a and b. Both hybridoma cell lines showed similar amounts of B cell receptors on their cell surfaces (approx. 1500 receptors per cell) as determined by flow cytometry (data not shown). Coupling SNAP-TTC to BG-647 did not affect the functionality of the protein, and we were able to detect specific binding of the protein to the surface of TTC-reactive hybridoma cells but not to the control hybridoma cells by flow cytometry (Fig. 3c and d). The TTC-ETA’ fusion protein exhibited a concentration-dependent binding to the TTC-reactive hybridoma cell line 5E4 compared to the control hybridoma cell line 8.18-C5 as shown in Fig. 4a and b.

### Viability of hybridoma cells following treatment with TTC fusion proteins

**Fig. 5:**
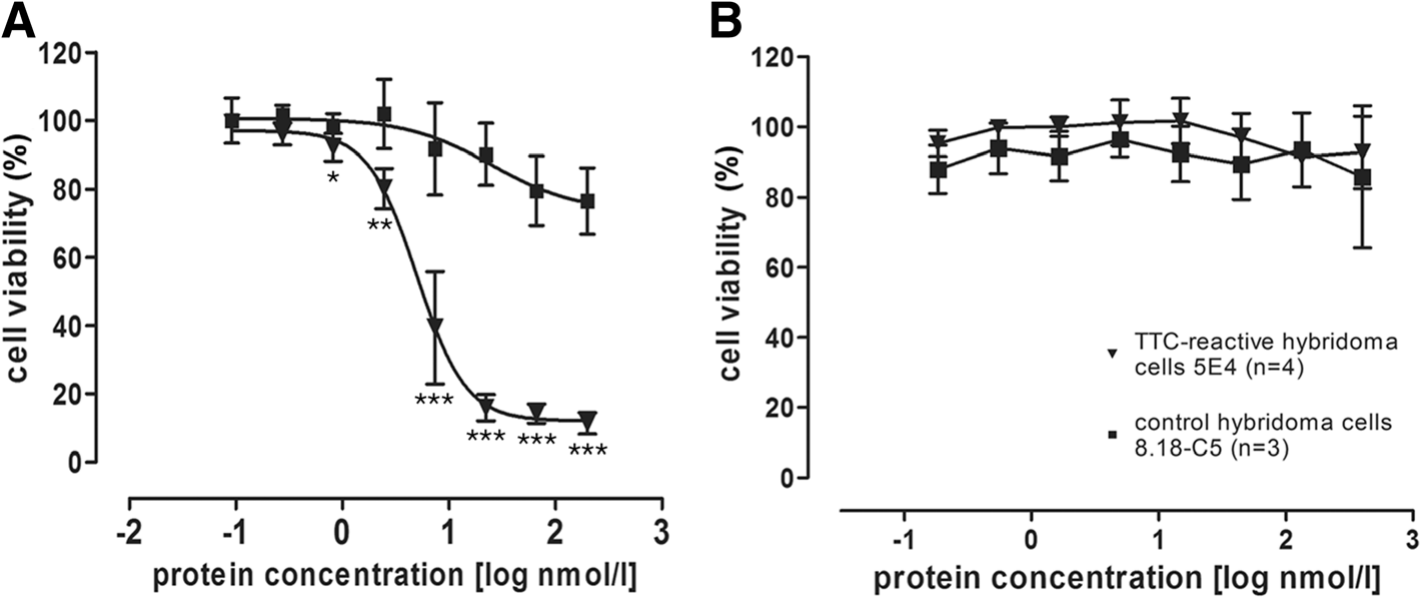
Cytotoxic activity of recombinant TTC-ETA’ on hybridoma cells. The hybridoma cell lines 5E4 (▼; *n* = 4) and 8.18-C5 (■; *n* = 3) were incubated with serial dilutions of TTC-ETA’ (**a**) and TTC (**b**) in complete RPMI 1640 cell culture medium. After incubation at 37 °C and 5 % CO_2_ for 72 h, the cells were refreshed with 50 μl RPMI 1640 medium containing XTT/phenazine methosulfate and incubated for another 4 h. Absorbance was measured at 450 nm and 630 nm on an ELISA reader. The half maximal inhibitory protein concentration (EC_50_) relative to untreated control cells was calculated using GraphPad Prism software. The recombinant TTC-ETA’ exhibits a dose-dependent cytotoxicity on the target hybridoma cell line 5E4 with an EC_50_of 1.3 nM ± 0.4 nM but has no effect on the control cell line 8.18-C5. These data represent the mean ± SD of three independent experiments performed in triplicates. *, *P* < 0.05; ** , *P* < 0.01; *P* < 0.001 versus control cells

After confirming the binding of the TTC fusion protein to the BCR on the surface of TTC-reactive 5E4 hybridoma cells, we investigated the toxicity of the TTC-ETA’ fusion protein in vitro using an XTT cell viability assay. TTC-ETA’ showed specific and dose-dependent toxicity towards TTC-reactive 5E4 hybridoma cells with a half inhibitory protein concentration (EC_50_) of 1.3 ± 0.4 nM, but they showed no toxicity towards control hybridoma cells (Fig. 5a). Furthermore, the TTC protein alone showed no toxicity towards either of the hybridoma cell lines (Fig. 5b).

### Binding analysis of TTC fusion proteins to B-lymphocytes

**Fig. 6:**
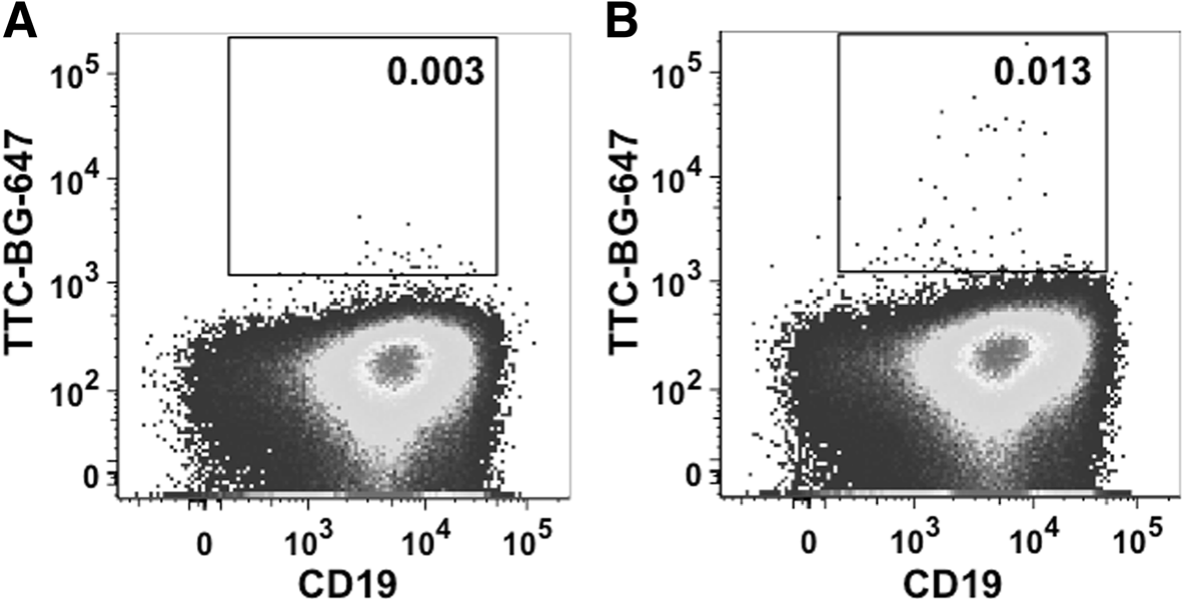
Binding analysis of recombinant SNAP-TTC-BG647 on polyclonally activated CD19^+^ B cells. CD19^+^ B cells were isolated from leukocyte filters by density gradient centrifugation and magnetic cell separation with anti-CD19 beads. Isolated B cells were activated by adding 2.5 μg/ml CpG and 50 ng/ml IL-21 to the cell culture medium. On day 4 of cultivation, the cells were analyzed by flow cytometry. Surface **a** and intracellular **b** staining with the recombinant SNAP-TTC-BG 647 (1:25) was performed

**Fig. 7:**
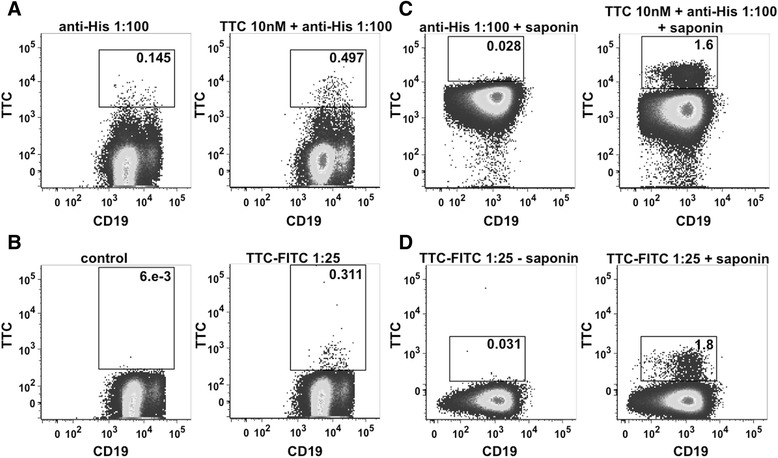
Binding analysis of recombinant TTC and TTC-FITC on isolated PBMCs. Surface staining of CD27^+^ memory B cells (**a**) and intracellular staining of CD27^++^CD38^++^ plasma cells (**c**) using recombinant TTC (10 nM) and anti-His5 Alexa Fluor 488 antibody (1:100). Surface staining of CD27^+^ memory B cells (**b**) and intracellular staining of CD27^++^CD38^++^ plasma cells (**d**) using a FITC-coupled TTC peptide (1:25)

Next, we determined the binding capacity of the SNAP-TTC-BG-647 protein to polyclonally activated CD19^+^ B cells isolated from leukocyte filters of vaccinated blood donors by flow cytometry. Figure 6 demonstrates that the frequencies of TTC-specific B cells remain low at day 4 after polyclonal activation (intracellular staining, Fig. 6b) compared to residual signals of the control B cells (surface staining, Fig. 6a). Since the frequencies of TTC-specific B cells were extremely low after polyclonal activation in donors without a recent booster vaccination (Fig. 6b), the binding of the TTC fusion protein was additionally verified on plasma cells and memory B cells isolated at day 6 and day 14 from a blood donor vaccinated recently, respectively. The samples were exposed to the recombinant TTC protein followed by an Alexa Fluor 488-conjugated anti-penta-his secondary antibody. Flow cytometry showed that tetanus-reactive CD27^+^ memory B cells (surface staining, Fig. 7a) as well as tetanus-reactive CD27^++^CD38^++^ plasma cells (intracellular staining, Fig. 7c) were detectable within the peripheral B cell pool shortly after booster vaccination. The frequencies of TTC-reactive memory B cells and plasma cells detected using the recombinant TTC protein was comparable to the use of the commercial FITC-conjugated TTC protein (Fig. 7b and d). TTC bound specifically to TTC-reactive memory B cells and plasma cells within the peripheral B cell pool.

### Cytotoxic activity of the recombinant TTC-ETA’ protein on polyclonally activated memory B cells

**Fig. 8:**
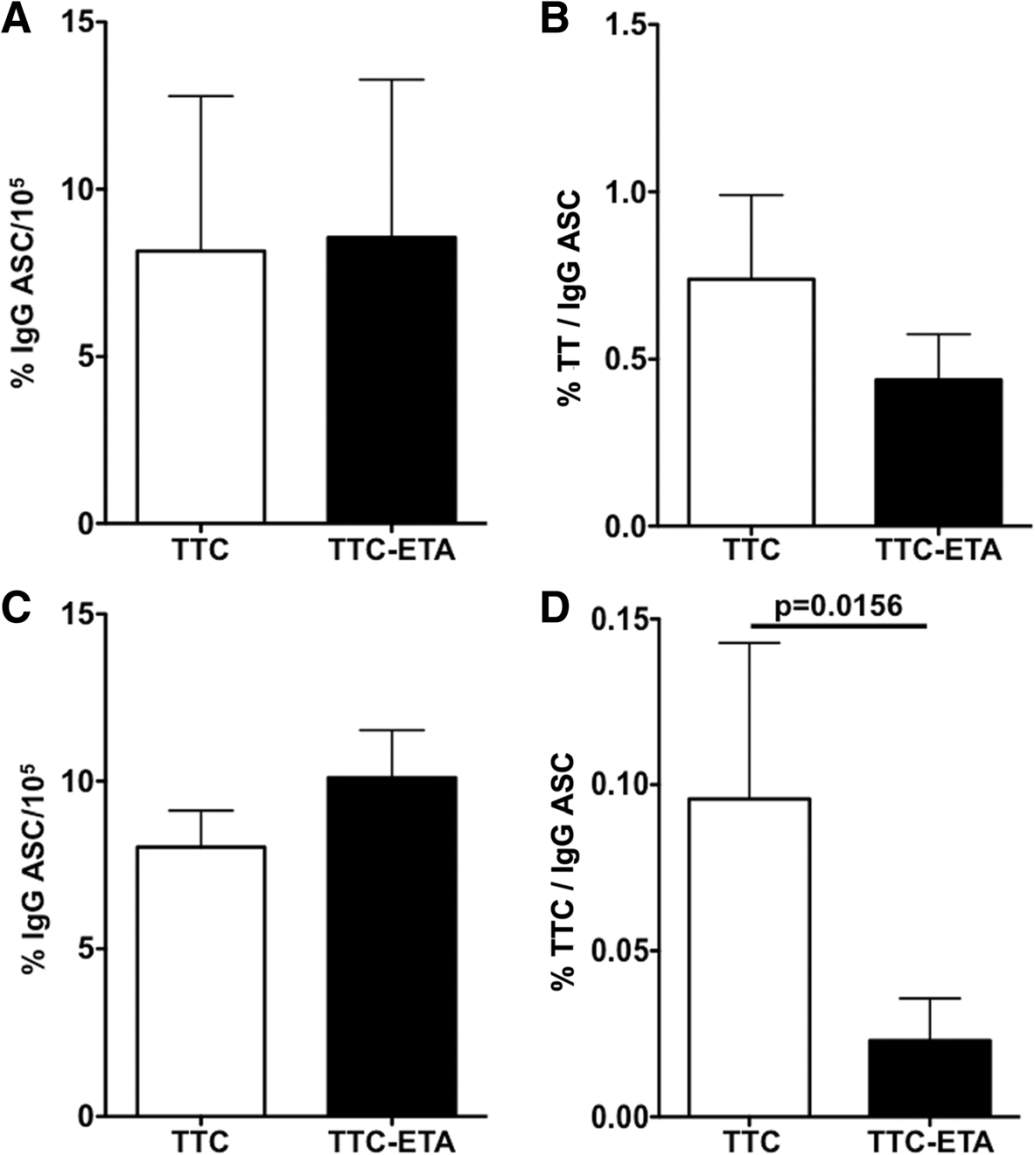
Cytotoxic activity of TTC-ETA’ on TTC-specific polyclonally activated memory B cells. Frequencies of IgG (**a**, **c**), of tetanus toxoid (TT)- (**b**) and of TTC-specific IgG (**d**) antibody-secreting cells (ASC) incubated with recombinant TTC (10 nM) or TTC-ETA’ (10 nM) protein on day 2 of polyclonal activation. The data are the results of 5 independent experiments using TT-coated plates (**b**) and **7** experiments using TTC-coated plates (**d**) and the corresponding total IgG ELISPOT assay (**a**, **c**). Statistical analysis was performed using the Wilcoxon matched-pairs signed rank test

Having confirmed the targeting of TTC-reactive memory B cells, we next set out to measure the depletion effects of the TTC-ETA’ protein. Therefore, CD27^+^ memory B cells were activated polyclonally to develop into antibody-secreting cells (ASCs) and were treated on day 2 of cultivation with 10 nM of recombinant TTC or TTC-ETA’. Next, ELISPOT analyses were performed to determine the frequency of all IgG-secreting cells as well as the TT-specific and TTC-specific IgG-secreting cells. The ASCs secreted IgG antibodies at comparable frequencies after treatment with TTC or TTC-ETA’ (Fig. 8a). The ELISPOT assays were carried out with commercial tetanus toxoid (TT) as the coating agent. When TTC protein was added to the cultures as the control protein, the ASCs secreted the TT-IgG antibodies at a frequency of 0.7 ± 0.6 % (Fig. 8b). Following the treatment with TTC-ETA’ the frequency of TT-IgG antibodies secreted by the ASCs declined to 0.4 ± 0.3 % (Fig. 8b). When tetanus toxoid fragment C (TTC) was used as the ELISPOT coating agent, we observed a small increase in the frequencies of general IgG antibodies following TTC-ETA’ treatment (Fig. 8c). The depletion effect of the TTC-ETA’ protein was confirmed by lower frequencies of secreted TTC-IgG antibodies decreasing significantly from 0.1 ± 0.12 to 0.02 ± 0.03 % (*p* = 0.0156) compared to treatment with the control protein TTC (Fig. 7d).

## Discussion

The bacterial pBM1.1 and the eukaryotic pMS expression vectors are platforms that allow the convenient insertion of sequences to genetically fuse antibody fragments or peptide-based binding moieties to cytotoxic effector domains. We were able to generate immunotoxin expression vectors with TTC as the cell-binding domain with or without ETA’ as the cytotoxic domain. TTC and TTC-ETA’ were expressed in bacteria and purified by IMAC, with a yield comparable to similar fusion proteins expressed in earlier investigations, such as MOG-ETA’ [10]. Periplasmic expression of fusion proteins in bacteria avoids the labor-intensive process of refolding proteins accumulated in inclusion bodies. Expression of the fusion proteins TTC and TTC-ETA’ was scalable, and under the applied osmotic stress conditions we found that the immunotoxin was more than 95 % functional [11, 20]. The TTC-reactive hybridoma cell line provides a simple and reliable test system for the TTC-ETA’ fusion protein as a “model immunotoxin”. The recombinant TTC-ETA’ protein demonstrated its functionality by specifically binding to its respective hybridoma cell line and displaying a dose-dependent cytotoxicity (Fig. 3). The SNAP-tag promotes the rapid and efficient coupling of reagents such as fluorescent dyes, and the resulting fluorescent SNAP-tag conjugates can be used as diagnostic tools because the function of the SNAP-tag fusion partner is not affected [17, 22, 23]. Here, we demonstrated the functionality of the SNAP-TTC-BG-647 protein by specific binding to TTC-reactive hybridoma (Fig. 3) and human memory B cells (Fig. 6) which was comparable to the binding activity of TTC and TTC-ETA'. Investigating TTC-specific ASCs after polyclonal activation of human memory B cells by ELISPOT we confirmed the specific binding of the TTC fusion proteins to CD27^+^ memory B cells isolated from human blood and their subsequent depletion resulting in a failure to generate TTC-specific ASCs after polyclonal activation and plasma cell differentiation. Compared to earlier developments by Volkman et al. [24] or Grailer et al*.* [25] we established an effective fusion protein expression platform enabling us to produce protein therapeutics in high yields and with homogeneous purity. Using this technology, we were able to show that a peptide fragment of an antigen recognized by the BCR can be used as the cell-binding component of a cytotoxic fusion protein, ensuring that the immunotoxin targets an antigen-specific B cell population without affecting the entire B cell pool. Both cell types (hybridoma cells and ASCs obtained from polyclonally activated memory B cells) produce soluble antibodies that can encounter and intercept the TTC fusion proteins in vitro, ex vivo and in vivo. Nevertheless, the TTC-ETA’ fusion protein showed dose-dependent cytotoxicity towards TTC-reactive 5E4 hybridoma cells with an EC50 value of 1.4 nM (Fig. 5). For TTC-ETA’, we determined a half maximal effective concentration (EC50) value that is comparable to related fusion proteins based on an allergen fragment [19] or a major autoantigen in multiple sclerosis [10]. The labeling of polyclonally activated CD19^+^ B cells by the SNAP-TTC-BG-647 protein showed that the number of tetanus-reactive B cells declines if a long time has elapsed since the last tetanus vaccination (Fig. 6). Self-reactive plasma cells are also rarely observed in the peripheral blood even in patients with active autoimmune disease [26] and data about self-reactive memory B cells in these patients is limited. Provided that their frequency is comparable with that of TTC-reactive B cells in vaccinated controls, it seems challenging to isolate and study these cells in patients with disease- or treatment-related lymphopenia. Hence, future investigations should consider strategies of polyclonal activation and subsequent enrichment of these memory B cells to raise their abundance. And for some autoantigens the use of animal models seems still inevitable. We found that exposing the activated human memory B cell pool to 10 nM TTC-ETA’ resulted in an 80 % inhibition of generated TTC-reactive IgG secreting cells compared to the control treatment with the TTC protein lacking a toxic domain (Fig. 8). These results clearly indicate that only the TTC-ETA’ fusion protein significantly affected the generation of TTC-reactive ASCs. Self-reactive memory B cells have been shown to be resistant to many standard therapeutic approaches [3]. They represent plasma cell precursors that are easy to activate [27].

We confirmed that it is possible to target antigen specific human memory B cells using novel cytotoxic fusion proteins containing part of the antigen as a cell-binding domain. This novel strategy will facilitate the development of targeted therapies based on the specific characteristics of each patient.

## Ethics statement

The materials and methods of this study were approved by the Ethics Committee of the General Medical Council Westfalen-Lippe and the Medical Faculty of Münster, Germany (approval number: 2009-413-f-S). The use of leukocyte filters, a by-product of blood donation was not considered to require written consent by the local IRB. The donors however gave written informed consent and were asked for their status of vaccination.

## Conclusions

This protein engineering strategy can be used as a generalized platform approach for the construction of therapeutic fusion proteins with disease-relevant antigens as B cell receptor-binding domains, offering a promising approach for the specific depletion of autoreactive B-lymphocytes in B cell-driven autoimmune diseases.

## Additional file

Additional file 1: Figure S1. Expression and purification of TTC-ETA' and TTC. Coomassie-stained SDS gel (left) and corresponding western blot (right) of TTC-ETA' (**1**; 95 kDa) and TTC (**2**; 55 kDa) produced by bacterial expression under osmotic stress conditions and purified by immobilized metal ion affinity chromatography and subsequent size exclusion chromatography. Proteins were separated by SDS polyacrylamide gel electrophoresis on a 12 % polyacrylamide gel and detected using a primary anti-penta-his antibody diluted (1:5000) and an alkaline phosphate (AP)-coupled goat-anti-mouse secondary antibody (1:5000). (TIF 10310 kb)

## Abbreviations

ADP: adenosine diphosphate
AMP: 2-amino-2-methyl-1-propanol
AP: alkaline phosphatase
ASC: antibody secreting cell
BCIP: 5-bromo-4-chloro-3'-indolyphosphate p-toluidine salt
BCR: b cell receptor
BG: benzylguanine
BSA: bovine serum albumin
CRi: Cambridge Research & Instrumentation
DAPI: 4',6-diamidino-2-phenylindole
DNA: deoxyribonucleic acid
*E.coli*: *Escherichia coli*
EC_50_: half maximal effective concentration
eEF2: eukaryotic elongation factor 2
ELISA: enzyme-linked immunosorbent assay
ELISPOT: enzyme-linked immunospot assay
ETA: *Pseudomonas aeruginosa* exotoxin A
FCS: fetal calf serum
FITC: fluorescein isothiocyanate
HEK: human embryonic kidney
IFN: interferon
Ig: immunoglobulin
IL: interleukin
IMAC: immobilized metal ion affinity chromatography
IRB: Institutional Review Board
kDa: kilodalton
Lf: limes flocculationis
MOG: myelin oligodendrocyte glycoprotein
MW: molecular weight
NBT: nitro-blue tetrazolium chloride
Ni-NTA: nickel nitrilotriacetic acid
PBMC: peripheral blood mononuclear cell
PBS: phosphate-buffered saline
PE: phycoerythrin
RPMI: Roswell Park Memorial Institute
SDS-PAGE: sodium dodecyl sulfate polyacrylamid gel electrophoresis
TGF: transforming growth factor
TLR: toll-like receptor
TTC: tetanus toxoid fragment C
XTT: 2,3-Bis-(2-Methoxy-4-Nitro-5-Sulfophenyl)-2H-Tetrazolium-5-Carboxanilide
