# Supplementary figures and images for: Novel fusion proteins for the antigen-specific staining and elimination of B cell receptor-positive cell populations demonstrated by a tetanus toxoid fragment C (TTC) model antigen
Source: BMC Biotechnol. 2016 Feb 17;16:18. doi: 10.1186/s12896-016-0249-x (PMC4756516; doi:10.1186/s12896-016-0249-x)

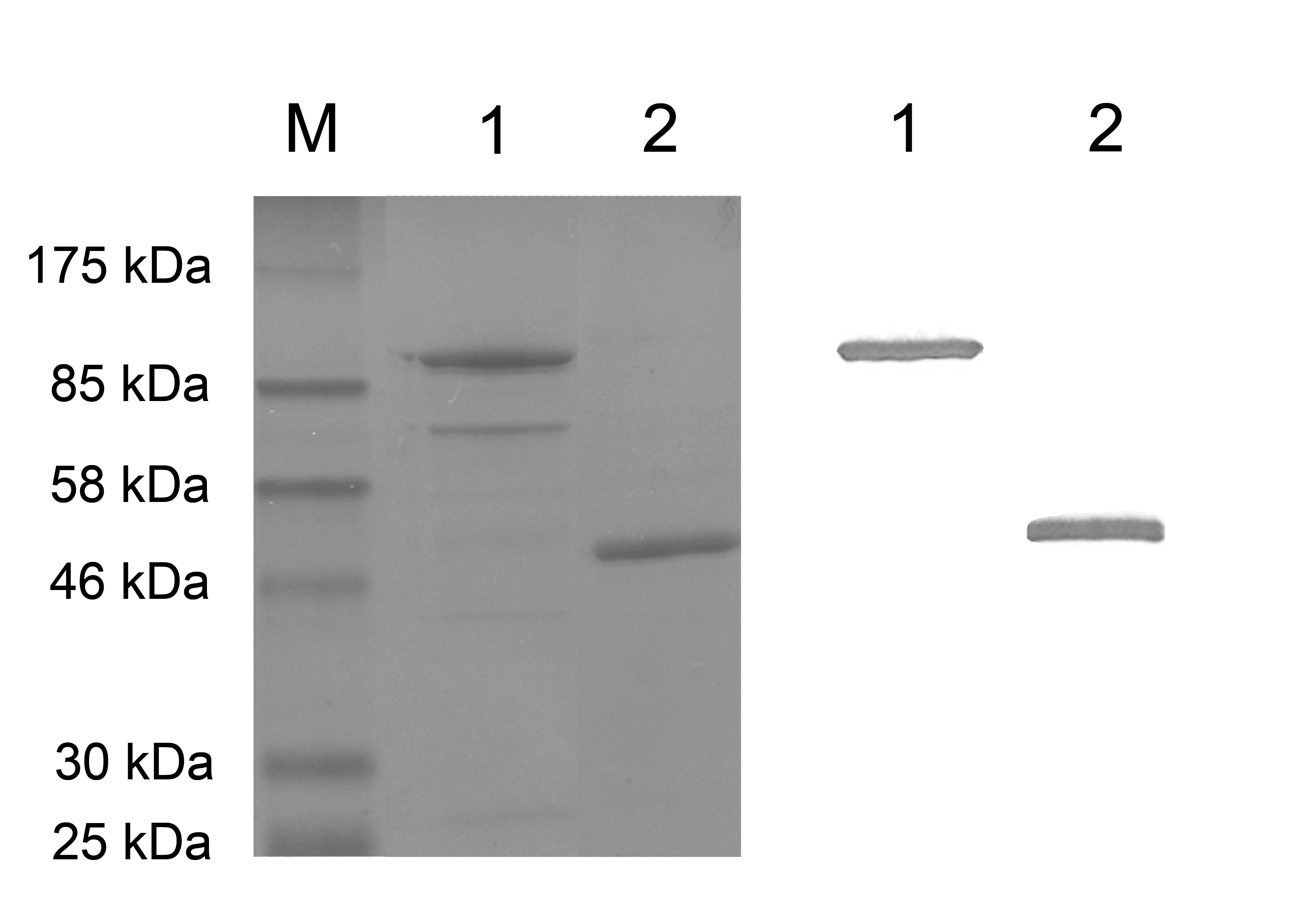

Supplement: Additional file 1: Figure S1. — Expression and purification of TTC-ETA' and TTC. Coomassie-stained SDS gel (left) and corresponding western blot (right) of TTC-ETA' (1; 95 kDa) and TTC (2; 55 kDa) produced by bacterial expression under osmotic stress conditions and purified by immobilized metal ion affinity chromatography and subsequent size exclusion chromatography. Proteins were separated by SDS polyacrylamide gel electrophoresis on a 12 % polyacrylamide gel and detected using a primary anti-penta-his antibody diluted (1:5000) and an alkaline phosphate (AP)-coupled goat-anti-mouse secondary antibody (1:5000). (TIF 10310 kb) [file 12896_2016_249_MOESM1_ESM.tif]
